# Procedure-Related Complications of Left Bundle Branch Pacing: A Single-Center Experience

**DOI:** 10.3389/fcvm.2021.645947

**Published:** 2021-03-24

**Authors:** Xueying Chen, Lanfang Wei, Jin Bai, Wei Wang, Shengmei Qin, Jingfeng Wang, Yixiu Liang, Yangang Su, Junbo Ge

**Affiliations:** ^1^Department of Cardiology, Zhongshan Hospital of Fudan University, Shanghai Institute of Cardiovascular Diseases, National Clinical Research Center for Interventional Medicine, Shanghai, China; ^2^Department of Cardiology, Xiamen Branch, Zhongshan Hospital, Fudan University, Xiamen, China

**Keywords:** left bundle branch pacing, His-Purkinje conduction system pacing, procedure-related complications, septal perforation, lead dislodgement, septum injury, lead fracture, safety

## Abstract

**Background:** Although left bundle branch pacing (LBBP) has emerged as a novel physiological pacing strategy with a low and stable threshold, its safety has not been well-documented. In the present study, we included all the patients with procedure-related complications at our centre to estimate these LBBP cases with unique complications.

**Methods:** We enrolled 612 consecutive patients who received the procedure in Zhongshan Hospital, Fudan University, between January 2018 and July 2020. Regular follow-ups were conducted (at 1, 3, and 6 months in the first year and every 6–12 months from the second year), and the clinical data of the patients with complications were collected and analyzed.

**Results:** With a mean follow-up period of 12.32 ± 5.21 months, procedure-related complications were observed in 10 patients (1.63%) that included two postoperative septum perforations (2/612, 0.33%), two postoperative lead dislodgements (2/612, 0.33%), four intraoperative septum injuries (4/612, 0.65%), and two intraoperative lead fractures (2/612, 0.33%). Pacing parameters were stable during follow-up, and no major complications were observed after lead repositioning in the cases of septum perforation and lead dislodgement.

**Conclusion:** The incidence of procedure-related complications for LBBP, namely postoperative septum perforation, postoperative lead dislodgement, intraoperative septum injury, and intraoperative lead fracture, were low. No adverse clinical outcomes were demonstrated after successful repositioning of the lead and appropriate treatment.

## Introduction

Left bundle branch pacing (LBBP) has emerged as a novel physiological pacing strategy with low pacing threshold and high R wave amplitude (1, 2). Several small size observational studies have reported that LBBP offers narrow QRS duration and superior mechanical synchrony (1–5). Moreover, the feasibility and efficacy of LBBP have also been demonstrated in candidates for cardiac resynchronisation therapy with heart failure and left bundle branch block (1, 6, 7). However, as a novel pacing technique, the safety of LBBP has not been well-documented. To capture the left conduction system, the LBBP lead should be screwed deep enough into the subendomyocardium of the left ventricle (8), which differs from the conventional right ventricular (RV) pacing lead. Consequently, unique procedure-related complications, such as interventricular septum perforation, lead dislodgement, septum injury, and lead fracture, of LBBP are observed. To date, only limited case reports (9, 10) and small observational studies (4, 5, 11) on these complications are available. However, these observations have been limited by indefinite criteria of LBBP, relatively short follow-up, and lack of the specific analysis of the complications. Therefore, in the present study, we attempted to collect and evaluate LBBP cases with unique complications from a consecutive large population in our center.

## Methods

### Study Population

The present retrospective single-centre observational study was conducted in all patients with procedure-related complications including septum perforation, lead dislodgement, septum injury and lead fracture from 612 consecutive patients who received LBBP in Zhongshan Hospital, Fudan University, between January 2018 and July 2020. Septum perforation was defined as the lead's tip penetrated the entire interventricular septum into the left ventricular cavity. While septum injury was defined as contrast agent retention during angiography through the delivery sheath. All the patients were discharged 1–2 days after the procedure in case of no evidence of complications, and they were asked to follow-up at 1, 3, and 6 months in the first year and every 6–12 months from the second year after the procedure for the assessment of device function and complications. Medical history, pacing parameters, 12-lead paced electrocardiogram, and fluoroscopic images of the patients with complications were recorded and analyzed. Written informed consent was obtained from all the enrolled participants, and the study was approved by the Institutional Review Board of Zhongshan Hospital, Fudan University, Shanghai, China.

### Implantation Procedure of LBBP

**Figure 1:**
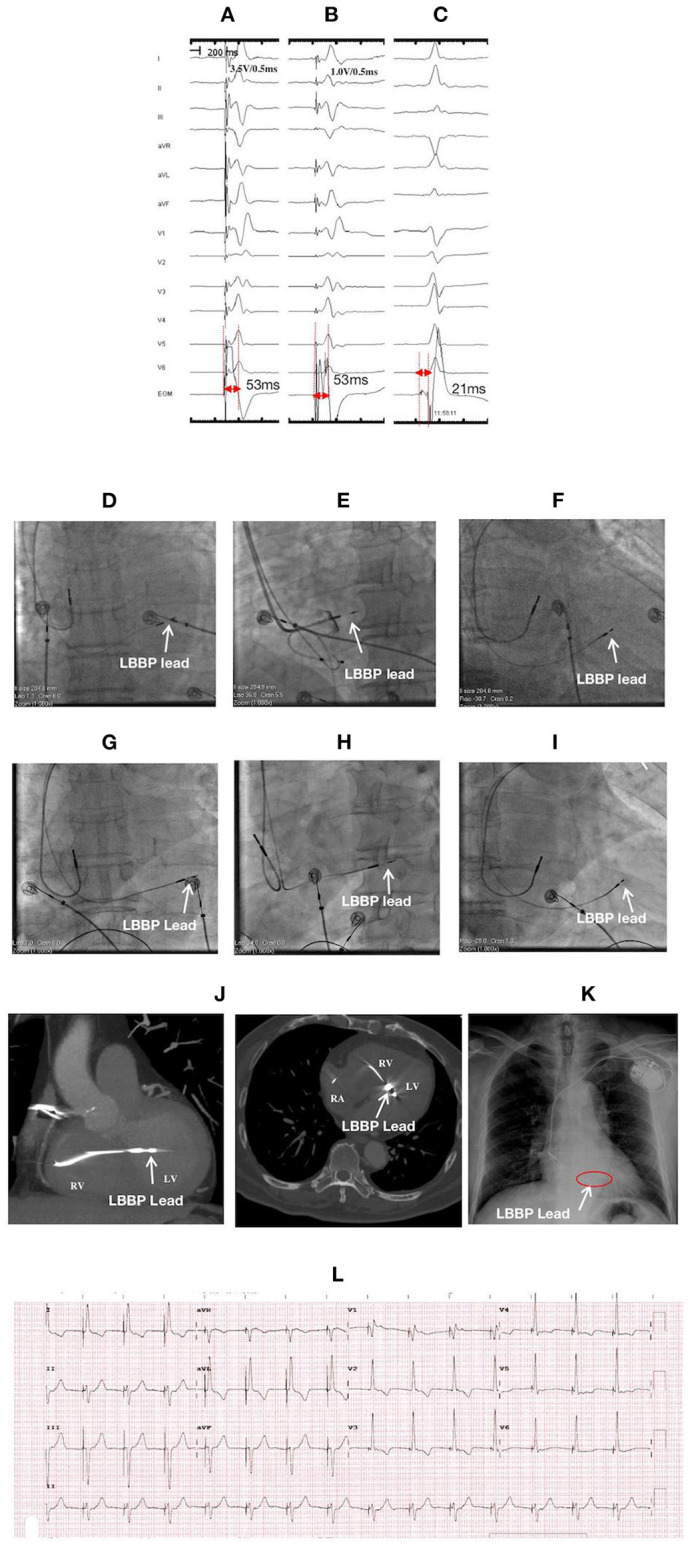
ECGs, EGMs, and fluroscopic images of a 78-year-old male with LBBP lead perforation to the left ventricular (LV) chamber at 1-month postoperative follow-up: During the first procedure: NSLBBP at 3.5 V/0.5 ms **(A)** and SLBBP at 1.0 V/0.5 ms **(B)** with the same Sti-LVAT of 53 ms, **(C)** Po_LBB_ during intrinsic rhythm with a Po_LBB_-V interval of 21 ms; Fluoroscopic images during the first procedure: **(D)** at PA, **(E)** at LAO 35° with angiography through the sheath exhibiting the LBBP lead depth inside the septum (white arrow), and **(F)** at RAO 30°; Fluoroscopic images before lead reposition: **(G)** at PA; **(H)** at LAO 35°, **(I)** at RAO 30°, and **(J)** CT imaging illustrating the lead perforation to LV chamber for approximately 1.5 cm (white arrow); After lead repositioning: **(K)** X-ray film illustrating lead location and **(L)** ECG. ECG, electrocardiogram; EGM, electrogram; LBBP, left bundle branch pacing; NSLBBP, nonselective left bundle branch pacing; SLBBP, selective left bundle branch pacing; Sti-LVAT, stimulus to left ventricular activivation time; PoLBB, left bundle branch potential; PoLBB-V, left bundle branch potential to ventricle; PA, posteroanterior; LAO, left anterior oblique; RAO, right anterior oblique; CT, Computed Tomography.

**Figure 2:**
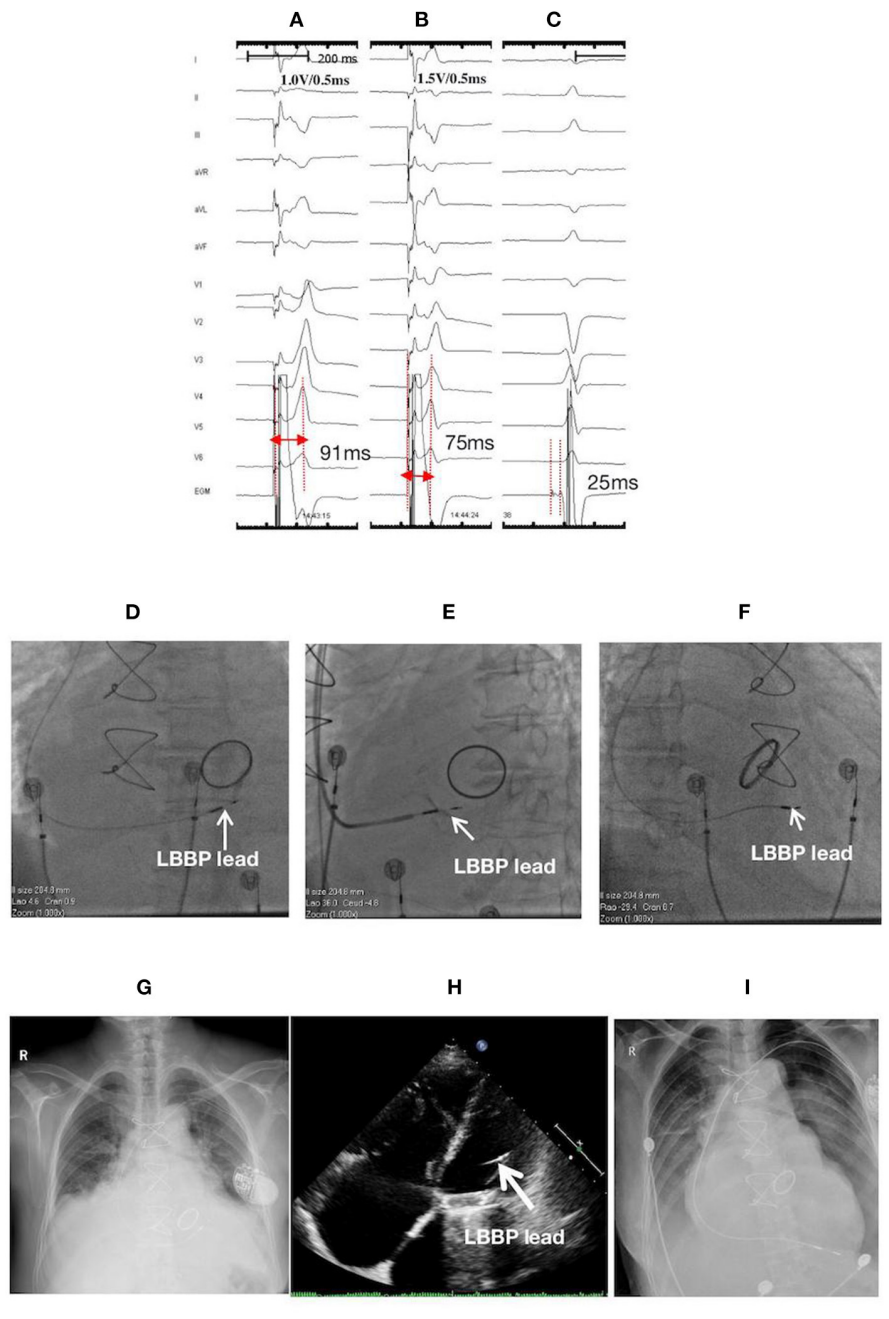
ECGs, EGMs, and fluoroscopic images of a 76-year-old female with LBBP lead perforation to the LV chamber on the second postoperative day: **(A)** LVSP at 1.0 V/0.5 ms with a Sti-LVAT of 91 ms; **(B)** NSLBBP at the same site with abrupt shortening of Sti-LVAT to 75 ms with increasing output (1.5 V/0.5 ms); **(C)** Po_LBB_ during intrinsic rhythm with a Po_LBB_-V interval of 25 ms; Fluoroscopic images during the first procedure: **(D)** at PA, **(E)** at LAO 35° with angiography through the sheath displaying the LBBP lead depth inside the septum (white arrow), and **(F)** at RAO 30°; At the second postoperative day: **(G)** X-ray film, and **(H)** Echocardiographic image illustrating lead perforation to the LV chamber (white arrow); **(I)** X-ray film after lead reposition to the RV apex. ECG, electrocardiogram; EGM, electrogram; LBBP, left bundle branch pacing; LV, left ventricle; LVSP, left ventricular septum pacing; NSLBBP, nonselective left bundle branch pacing; Sti-LVAT, stimulus to left ventricular activivation time; Po_LBB_, left bundle branch potential; Po_LBB_-V, left bundle branch potential to ventricle; PA, posteroanterior; LAO, left anterior oblique; RAO, right anterior oblique; RV, right ventricle.

The LBBP was performed according to the procedure described in literature (1, 2, 11). The pacing lead (Model 3830 69-cm, Medtronic, Minneapolis, USA) and C315 His sheath were used to map the potential of the His bundle by connecting the lead to an electrophysiology (EP) recording system (GE CardioLab EP Recording System 2000 GE Inc. Wisconsin, USA). Then the lead was placed 1–2 cm distal the His bundle location and in the direction of the RV apex under the fluoroscopic image of the right anterior oblique (RAO) 30 degree. The lead was screwed deeply into the interventricular septum until the paced QRS complex changed from an LBBB to a RBBB morphology. LBB capture was confirmed using RBBB paced morphology and one of the following signs: (1) selective LBBP (SLBBP) (paced morphology as a typical RBBB shape with a discrete component in intracardiac electrogram); (2) stimulus to left ventricular activation time (Sti-LVAT) shortening abruptly by >10 ms with increasing output or remaining shortest and constant at the final site (2, 12–14). When LBB capture threshold was lower than the local myocardium capture threshold, SLBBP could be achieved at low output while nonselective LBBP (NSLBBP) at high output (Figures 1A,B). On the contrary, when LBB capture threshold was higher than that of the myocardium, abrupt shortening of Sti-LVAT by >10 ms could be achieved by increasing output at the same site with left ventricular septum pacing (LVSP) at low output and NSLBBP at high output (Figures 2A,B). The LBB capture threshold ≤ 1.5 V/0.5 ms was recognized as acceptable (12).

### Statistical Methods

Continuous variables were reported as means ± standard deviation (SD) and compared by Student's *t*-test. Categorical variables were expressed as percentages and compared by using Pearson's χ2 test. *P*-values < 0.05 was considered statistically significant. All analyses were done by SPSS version 17 (SPSS Inc., Chicago, IL, USA).

## Results

Of the 612 patients who received LBBP at our center, with a mean follow-up of 12.32 ± 5.21 months, procedure-related complications were observed in 10 patients (1.63%); the complications included two postoperative septum perforations (2/612, 0.33%), two postoperative lead dislodgements (2/612, 0.33%), four intraoperative septum injuries (4/612, 0.65%), and two intraoperative lead fractures (2/612, 0.33%). The characteristics at baseline between the LBBP cases with and without complications were not significantly different (Table 1). During the procedure, there was no significant difference concerning the percentage of SLBBP in cases with and without complications (80.00 vs. 71.43%, *P* = 0.733) (Table 1). After lead repositioning in cases of postoperative septum perforation and lead dislodgement, pacing parameters were stable during follow-up, and no major complications such as transient ischemic attack or stroke, thrombus, infection, ventricular septal defect, and pericardial effusion were observed.

**Table 1:** Comparisons between LBBP with and without complications.

	**LBBP without complications (*n* = 602)**	**LBBP with complications (*n* = 10)**	***P*-value**
Age	70.08 ± 10.21	72.90 ± 6.81	0.385
Female	289 (48.00)	4 (40.00)	0.754
Hypertension	256 (42.53)	5 (50.00)	0.751
Diabetes	72 (11.96)	1 (10.00)	0.346
Atrial fibrillation	92 (15.28)	2 (20.00)	0.656
Pacemaker types			0.062
Single chamber pacemaker, *n* (%)	138 (22.92)	4 (40.00)	
Dual chamber pacemaker, *n* (%)	288 (47.84)	6 (60.00)	
CRT/CRTD	176 (29.23)	0 (0.00)	
Pacemaker indication			0.093
SSS, *n* (%)	116 (19.27)	3 (30.00)	
AVB, *n* (%)	280 (46.51)	6 (60.00)	
Heart failure indicated for CRT/CRTD	176 (29.23)	0 (0.00)	
Atrial fibrillation with low ventricular rate, *n* (%)	30 (4.98)	1 (10.00)	
SLBBP (%)	430 (71.43)	8 (80.00)	0.733

### Postoperative Septum Perforation

Of the 612 patients, two patients with postoperative septum perforation (one at the second day and one at 1 month) were observed (Table 2). Details of the cases are described as follows:

**Table 2:** Septum perforation and lead dislodgement cases.

**Case No**.	**Age**	**Gender**	**Diagnosis**	**Complication**	**Abnormal pacing parameters**	**Treatment and outcome**
1	78	Male	Sick sinus syndrome with paroxysmal atrial fibrillation	Septum perforation at 1-month follow-up	Threshold: >5.0 V/0.5 ms (unipolar) 2.5 V/0.5 ms (bipolar) Impedance: <300 Ω (unipolar)	The lead was repositioned to a more distal LBB area at posterior septum
2	76	Female	Atrial fibrillation with low ventricular rate	Septum perforation at the second day post-procedure	loss of capture at the high output (>7.5 V/0.5 ms)	A new lead (Model 5076) was implanted and replaced at RV apex
3	77	Female	Complete AVB	Lead dislodgement	loss of capture at the high output (>7.5 V/0.5 ms)	The lead was replaced to another LBB region with proper slack
4	64	Male	Complete AVB and atrial fibrillation	Lead dislodgement at 1-month follow-up and the lead dislodgement occurred again at 5-month after reposition.	loss of capture at the high output (>7.5 V/0.5 ms)	The lead was replaced to another LBB region but dislodged again Finally another lead (Model 5076) was repositioned at RV septum

#### Case 1

A 78-year-old male received LBBP due to sick sinus syndrome with paroxysmal atrial fibrillation. Nonselective LBBP (NSLBBP) and selective LBBP (SLBBP) were achieved at different outputs during the procedure, and the LBB potential (Po_LBB_) was mapped (Figures 1A–C). The pacing parameters were normal, and the angiography through the sheath revealed the lead depth inside the septum (Figure 1E). At the 1-month postoperative follow-up, the pacing threshold of the LBBP lead increased dramatically (>5.0 V/0.5 ms during unipolar pacing and 2.5 V/0.5 ms during bipolar pacing), and impedance reduced to <300 Ω during unipolar pacing. Computed tomography (CT) imaging and echocardiogram demonstrated LBBP lead perforation into the left ventricular cavity for ~1.5 cm (Figure 1). The lead was repositioned to a more distal LBB area at the posterior septum with confirmation of LBB capture and paced QRS with left axis deviation. The pacing parameters were stable at the 1-year follow-up.

#### Case 2

A 76-year-old female with low body mass index (BMI) (18.02 kg/m^2^) and having atrial fibrillation with low ventricular rate and dilated atrium received LBBP with a single-chamber pacemaker. The screwing of the lead deep inside the septum was challenging in this case probably due to lack of support from the sheath. After multiple attempts, the lead was finally screwed into the LBB area by using the “sheath in sheath” technique (C315 His sheath in CS sheath) (Figures 2D–F). The thresholds of the left ventricular septal pacing (LVSP) and nonselective LBBP were 1.0 V/0.5 ms and 1.5 V/0.5 ms, with Sti-LVAT of 91 and 75 ms, respectively (Figures 2A–C). Septum perforation was demonstrated through X-ray film, echocardiogram (Figures 2G,H), and loss of capture at high output (>7.5 V/0.5 ms) during both unipolar and bipolar pacing at the second postoperative day. The lead was withdrawn, and a new lead (Model 5076, Medtronic, Inc.) was implanted and repositioned at the RV apex (Figure 2I).

### Postoperative Lead Dislodgement

Of the 612 cases who received LBBP, two cases of postoperative lead dislodgement were observed [one at 1 month, and one at 1 month with recurrence of dislodgement 5 months after repositioning (Table 2)].

#### Case 3

**Figure 3:**
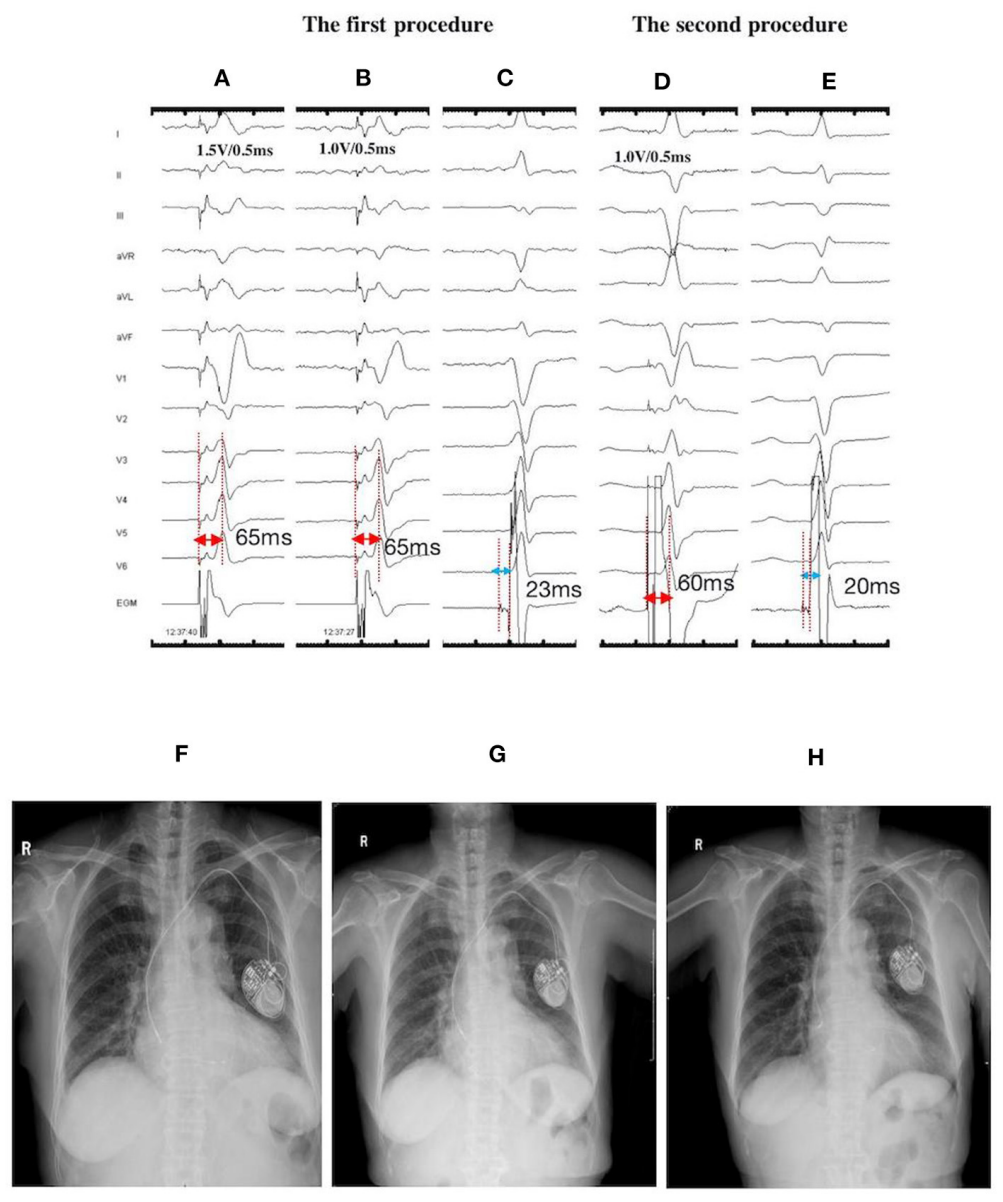
ECGs, EGMs, and fluoroscopic images of a 77-year-old female with LBBP lead dislodgement at the 1-month postoperative follow-up: During the first procedure: NSLBBP at 1.5 V/0.5 ms **(A)** and SLBBP at 1.0 V/0.5 ms **(B)** with the same Sti-LVAT of 65 ms; **(C)** Po_LBB_ during intrinsic rhythm with the Po_LBB_-V interval of 23 ms; During the second procedure: **(D)** NSLBBP at 1.0 V/0.5 ms, with the Sti-LVAT of 60 ms; **(E)** Po_LBB_ during intrinsic rhythm, with the PoLBB-V interval of 20 ms; X-ray films illustrating lead locations: **(F)** on the second day after the first procedure, **(G)** at the 1-month follow-up exhibiting lead dislodgement, and **(H)** on the second day after lead repositioning. ECG, electrocardiogram; EGM, electrogram; LBBP, left bundle branch pacing; NSLBBP, nonselective left bundle branch pacing; SLBBP, selective left bundle branch pacing; Sti-LVAT, stimulus to left ventricular activivation time; Po_LBB_, left bundle branch potential; Po_LBB_-V, left bundle branch potential to ventricle.

A 77-year-old female with complete atrioventricular block (AVB) received LBBP with a dual-chamber pacemaker. The LBBP was confirmed by achieving NSLBBP and SLBBP at different outputs, with a constant Sti-LVAT of 65 ms and recording Po_LBB_ (Figures 3A–C). The X-ray film taken before discharge displayed less slack. However, the pacing parameters were stable. Lead dislodgement was confirmed by a high pacing threshold (>7.5 V/0.5 ms) and through X-ray film at the 1-month follow-up (Figure 3G). The lead was repositioned to another LBB region with appropriate slack (Figures 3D,E,H), and the pacing parameters were stable at the 1-year follow-up.

#### Case 4

**Figure 4:**
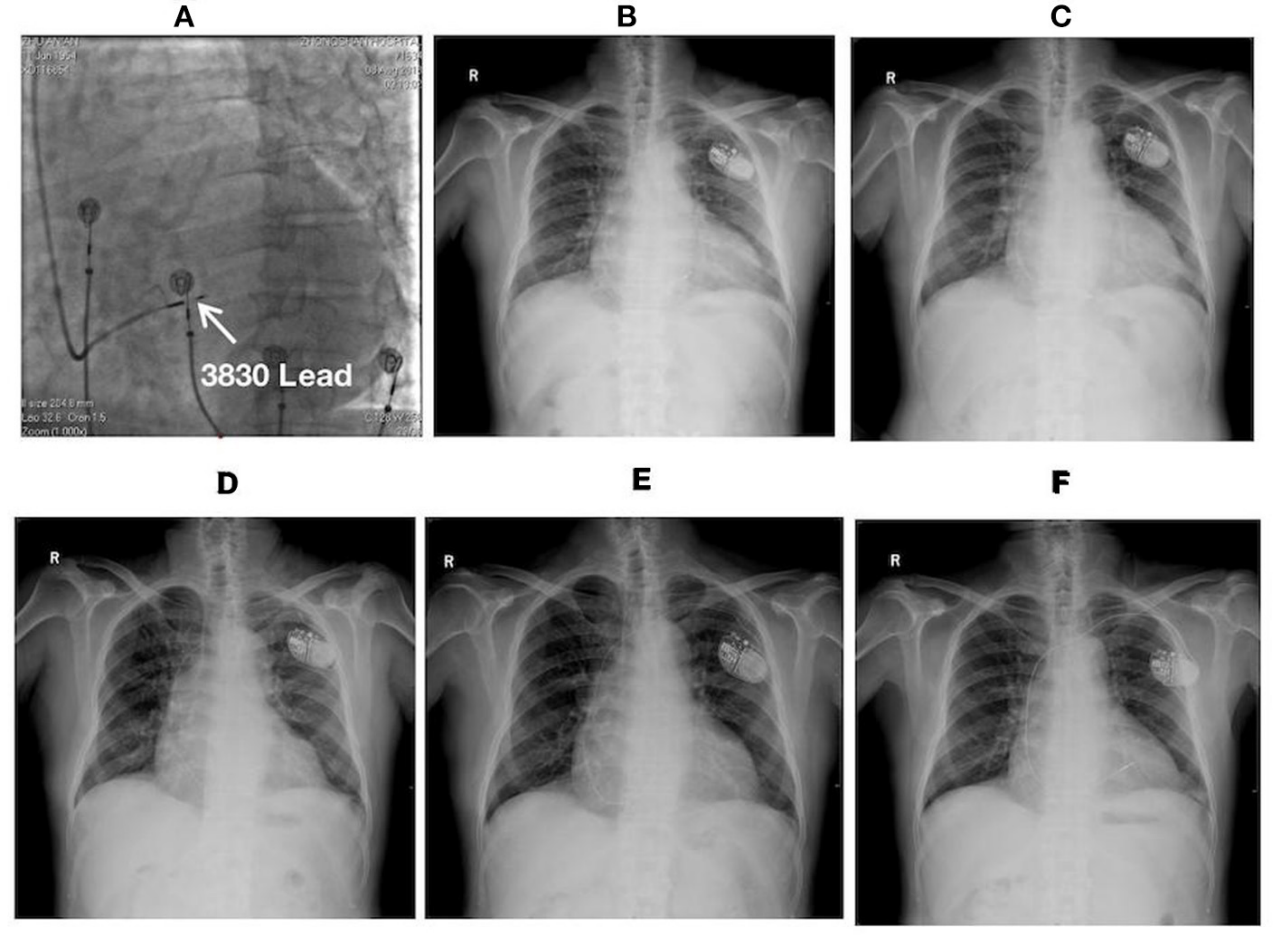
Fluoroscopic images of a 64-year-old male with LBBP lead dislodgement: **(A)** The fluoroscopic image at LAO 30° during the procedure illustrating the lead depth inside the septum (white arrow); **(B)** Postoperative X-ray film; **(C)** X-ray film at the 1-month postoperative follow-up illustrating lead dislodgement; **(D)** X-ray film after lead reposition; **(E)** X-ray film at 5 months after lead reposition demonstrating the recurrence of lead dislodgement; **(F)** X-ray film on the 2nd day after the 2nd lead repositioning demonstrating the repositioning of a new lead (Model 5076, Medtronic Inc., Minneapolis, MN, USA) at the RV septum. LAO, left anterior oblique.

A 64-year-old male with complete AVB and atrial fibrillation received LBBP with a single-chamber pacemaker. The echocardiogram displayed enlargement of the right atrium (78 × 69 mm) and increase in diameter of the basal segment of the right ventricle (49 mm) with severe tricuspid regurgitation. LBBP was finally achieved with optimum pacing parameters after multiple attempts. At 1-month after the procedure, the lead dislodgement to the RV apex was confirmed by a high pacing threshold (>7.5 V/0.5 ms) and through X-ray film (Figure 4). The lead was repositioned to another LBB region with superior pacing threshold and R wave amplitude. However, the pacing impedance was relatively low (~300–400 Ω). Pacing parameters remained stable until 2-months after the procedure. Lead dislodgement recurred 5 months after repositioning. The lead was withdrawn, and another lead (Model 5076, Medtronic Inc., Minneapolis, MN, USA) was repositioned at the RV septum. The pacing parameters remained stable afterwards.

### Intraoperative Septum Injury

**Figure 5:**
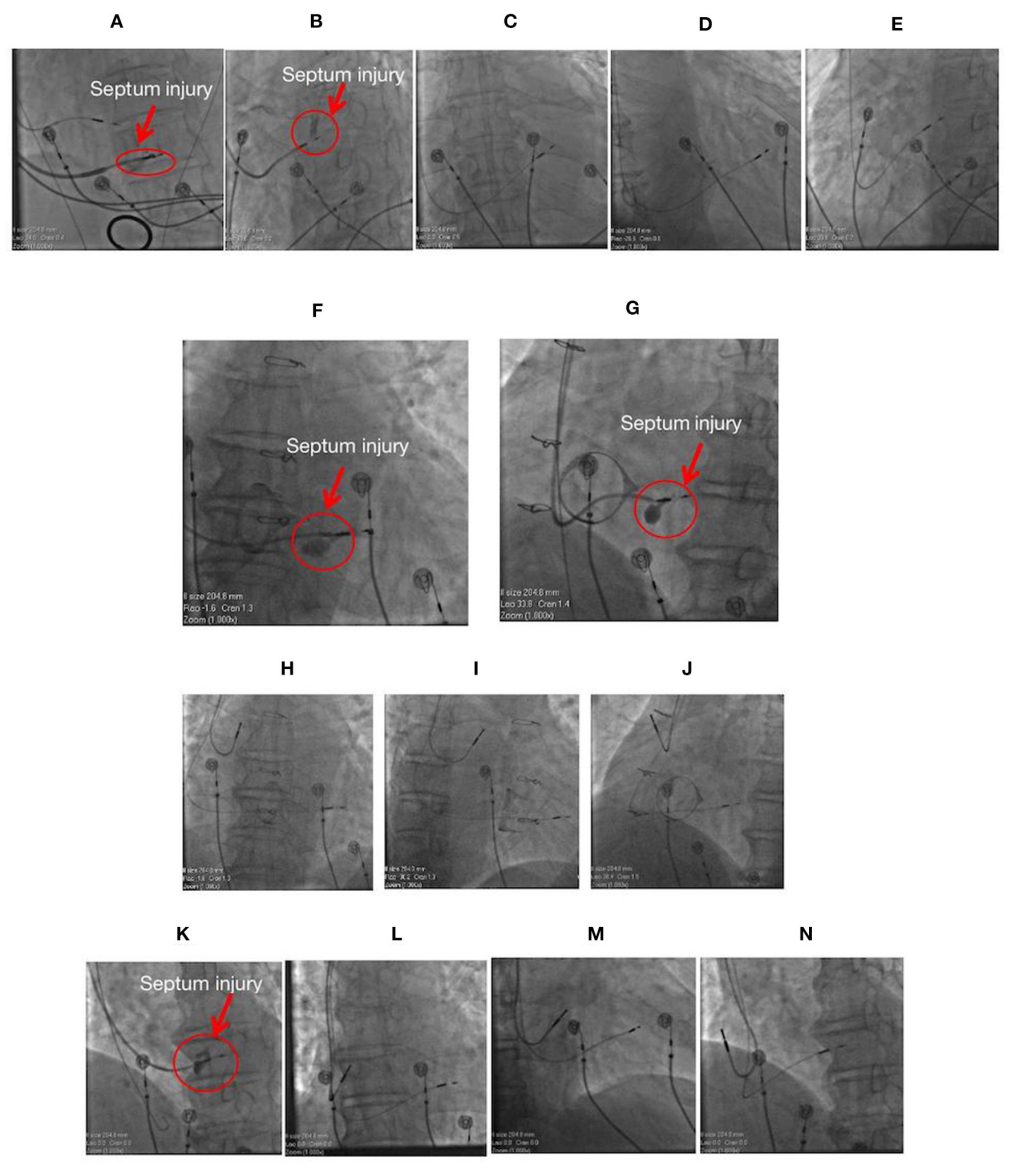
Images of four cases with septum injury during the procedure. **(A)** The fluoroscopic image of a 71-year-old female having mitral regurgitation and sick sinus syndrome with ventricular septum injury (red arrow) during LBBP lead implantation at LAO 35°. **(B)** The fluoroscopic image of a 77-year-old male with atrial fibrillation and complete AVB with septum injury (red arrow) during LBBP lead implantation with a single-chamber pacemaker at LAO 35°. The lead was not repositioned, and its location is illustrated at PA **(C)**, RAO30° **(D)**, and LAO 35° **(E)**. Fluoroscopic images of a 64-year-old male with sick sinus syndrome post-bioprosthetic tricuspid valve replacement having septum injury (red arrow) during LBBP lead implantation with a dual-chamber pacemaker at PA **(F)** and LAO 35° **(G)**. The lead was not repositioned, and its location is illustrated at PA **(H)**, RAO30° **(I)**, and LAO 35° **(J)**. **(K)** The fluoroscopic image of an 85-year-old male with complete AVB and atrial fibrillation having septum injury (red arrow) during LBBP lead implantation with a dual-chamber pacemaker at LAO 35°. The lead was not repositioned, and its location is illustrated at PA **(L)**, RAO30° **(M)**, and LAO 35° **(N)**. LBBP, left bundle branch pacing; AVB, atrioventricular block; PA, posteroanterior; LAO, left anterior oblique; RAO, right anterior oblique.

Of the 612 cases, four cases of intraoperative ventricular septum injury were identified (Figure 5). Approximately 5 mL of contrast agent was injected through the delivery sheath (C315 His; Medtronic Inc., Minneapolis, MN, USA) and placed close to the right side of the interventricular septum to determine the exact depth of the lead inside the septum after the lead was confirmed to have achieved LBBP. High pressure was determined during the contrast injection in these four cases, and the contrast agent retention was recorded to detect intraoperative septum injury. The patients did not complain of any symptoms such as chest pain and shortness of breath, and the electrocardiogram did not exhibit ST-segment elevation or depression in any leads. Pacing parameters were measured several times and were found to be stable after contrast injection. No obvious septal abnormalities were observed in the echocardiogram of the four cases during procedure. LBBP leads of all four cases were not repositioned afterwards. Cardiac troponin T (CTNT) levels on the second postoperative day were found to be mildly elevated compared with the preoperative levels (Table 3). During the follow-up, no evidence of myocardial infarction, septum perforation, and lead dislodgement was identified, and pacing parameters remained acceptable and stable (Table 3).

**Table 3:** Characteristics of four cases with septum injury during procedure.

**Case No**.	**Age**	**Gender**	**Diagnosis**	**CTNT level (ng/ml) post-procedure**	**CTNT level (ng/ml) at 2rd day post-procedure**	**Pacing parameters (unipolar) during procedure**	**Pacing parameters (unipolar) during follow-up**
1	71	Female	Sick sinus syndrome	0.084	0.143	Threshold: 1.0 V/0.5 ms R wave amplitude: 10 mV Impedance: 510 Ω	Threshold: 0.75 V/0.5 ms R wave amplitude: 12 mV Impedance: 436 Ω (at 18-month follow-up)
2	77	Male	Atrial fibrillation with AVB	0.015	0.05	Threshold: 0.8 V/0.5 ms R wave amplitude: 15 mV Impedance: 620 Ω	Threshold: 0.5 V/0.5 ms R wave amplitude: 14 mV Impedance: 490 Ω (at 18-month follow-up)
3	66	Male	Sick sinus syndrome	0.01	0.07	Threshold: 0.5 V/0.5 ms R wave amplitude: 17 mV Impedance: 464 Ω	Threshold: 0.5 V/0.5 ms R wave amplitude: 15 mV Impedance: 386 Ω (at 24-month follow-up)
4	85	Male	Atrial fibrillation with AVB	0.065	0.074	Threshold: 0.8 V/0.5 ms R wave amplitude: 5 mV Impedance: 680 Ω	Threshold: 0.75 V/0.5 ms R wave amplitude: 8 mV Impedance: 512 Ω (at 12-month follow-up)

### Lead Fracture

**Figure 6:**
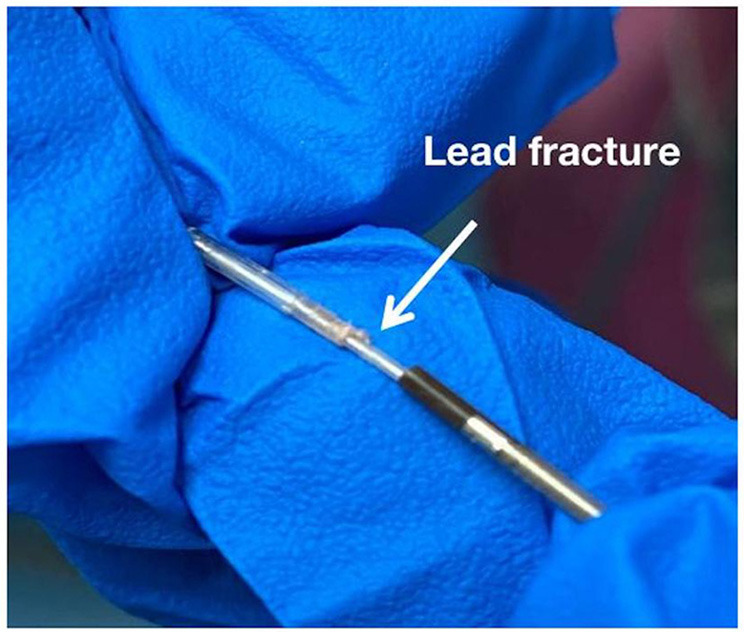
Image of lead fracture between the lead body and the end of the ring.

Of the 612 cases, two cases of LBBP lead (model 3830, 69 cm; Medtronic, Inc.) fracture were identified during the procedure when it was hard to advance the leads. After multiple attempts, the leads were withdrawn, and disconnection between the lead body and end of the ring was demonstrated (Figure 6). The lead was subsequently abandoned, and new leads were implanted to another site to achieve LBBP. Figure 6 represent the image of lead fracture between the lead body and the end of the ring.

## Discussion

LBBP is an emerging alternative physiological technique to His bundle pacing. Although the definitions and characteristics of this procedure have been established and its short-term safety profile has been demonstrated, the long-term safety remains unknown. In the present study, we demonstrated the possible procedure-related LBBP complications, including ventricular septum perforation (two cases), lead dislodgement (two cases), septum injury (four cases), and lead fracture (two cases) in a relatively large population during a mean follow-up of 12.32 ±5.21 months.

### Lead Dislodgement and Septum Perforation

Of the 530 published cases in literature, 6 (1.1%) lead dislodgements (one intraoperative, three within 24 h, one at 2 months, and one at 4 months) and 9 (1.7%) septal perforations (eight intraoperative and one at 1 month) have been identified (4, 5, 15–17). In the present study, two cases of lead dislodgement were identified by the high threshold, low impedance, and X-ray film at the 1-month follow-up. One of these two cases received LBBP lead replacement to a more distal LBB site, and the pacing parameters were stable during follow-up. LBB is a wide network beneath the endomyocardium of the left septum (18). Thus, positioning of the lead at this area could easily capture the left conduction system (8). In case of lead dislodgement and perforation, repositioning of the lead to a distal LBB area could probably prevent the recurrence of these complications because the original area might be injured by the lead, and the fixation of lead in posterior septum through the C315 His sheath is simple. Similar to the conventional RV lead, the risk of myocardial perforation would be high for an older woman with low BMI (19). In case 2, the large atrium might have lead to heart transposition, which causes difficulties in screwing the lead into the septum. Multiple attempts might cause injury to the septum and increase the risk of perforation. Additionally, heart contraction is an axial twisting movement (20). The torsion between the large atrium and septum might result in perforation after removal of the sheath. This might be a possible explanation for the recurrence of lead dislodgement after lead repositioning to another LBB area. The lead dislodgement in case 3 might be attributed to less slack (Figure 3F). Proper slack is crucial to acute and chronic lead dislodgement and perforation.

### Septum Injury

Multiple attempts at positioning the lead inside the septum and the procedure of the contrast injection itself might be the possible causes of septum injury, and these were the probable reason of septum injury of the four cases in the present study. During the contrast injection, the sheath should be pulled slightly backwards from the right septum to avoid septum injury due to the sheath or pressure of contrast injection. Under this circumstance, the repositioning of lead to another site might not be required, if the pacing parameters remain stable and no evidence of myocardial ischaemia is observed.

### Lead Fracture

The LBBP lead must be screwed deep enough into the subendomyocardium of the left septum. VJ et al. reported that the average lead depth inside the septum is 1.4 ± 0.23 cm (5), whereas the helix length of the lead is only 1.8 mm, which is designed for conventional RV pacing. To reach the desired LBB area, the lead needs to be screwed at least 10 turns, which is much more than that recommended by the manufacturer (4–6 turns). Thus, the possibility of lead fracture would be higher than than that with conventional RV pacing. We observed two cases of intraoperative lead fracture and successfully performed LBBP with another new lead. Lead check should be considered, if screwing of the lead during the procedure becomes difficult. Long-term lead performance of LBBP has not been demonstrated yet. Thus, to identify chronic lead fracture during follow-up, a more frequent lead check than the conventional RV pacing in LBBP is recommended.

Although LBBP complications may occur intraoperatively or postoperatively, the incidence is slightly low in the present study (1.63%). No adverse clinical outcomes were demonstrated with these complications after appropriate treatment. However, due to lack of long-term follow-up, the complications should be carefully detected both intraoperatively and postoperatively. Evaluation of the preoperative septal thickness and characteristics, minimization of multiple attempts in the same region, adequate lead slack, and frequent follow-ups could be helpful in avoiding complications. Postoperative follow-up, particularly more frequent monitoring, could also help in promptly detecting possible complications and administering clinical interventions as early as possible to avoid adverse outcomes.

### Limitation

The present study was a retrospective observational study performed at a single centre with a short- to mid-term follow-up. The follow-up period was not sufficiently long to draw a conclusion on the long-term safety of LBBP. Lead performance during long-term follow-up is unknown at present. Moreover, operator experience might influence the incidence of complications. Consequently, long-term, multi-centre, case–control, and randomized trials are required to confirm the safety of LBBP relative to the conventional ventricular pacing.

## Conclusion

The incidence of procedure-related LBBP complications including postoperative septum perforation, postoperative lead dislodgement, intraoperative septum injury, and intraoperative lead fracture was low. Additionally, no adverse clinical outcomes of these complications were observed after successful repositioning of leads and appropriate treatment.

## Data Availability Statement

The datasets generated during and analyzed during the current study are available from the corresponding author on reasonable request.

## Ethics Statement

This study is a retrospective single-center study. Written informed consent was obtained from the individual(s) to publish any potentially identifiable images or data included in this article. All patients signed informed consent to participate in the study.

## Author Contributions

XC, YS, and JG designed the research. XC, JB, WW, JW, SQ, and YL performed operations on the above-mentioned patients. XC and LW collected and analyzed data and wrote the papers. All authors contributed to the article and approved the submitted version.

## Conflict of Interest

The authors declare that the research was conducted in the absence of any commercial or financial relationships that could be construed as a potential conflict of interest.
